# Coverage and Prior Authorization Policies for Medications for Opioid Use Disorder in Medicaid Managed Care

**DOI:** 10.1001/jamahealthforum.2022.4001

**Published:** 2022-11-04

**Authors:** Amanda J. Abraham, Christina M. Andrews, Samantha J. Harris, Melissa M. Westlake, Colleen M. Grogan

**Affiliations:** Department of Public Administration and Policy, University of Georgia School of Public and International Affairs, Athens; Arnold School of Public Health, Health Services Policy and Management Department, University of South Carolina, Columbia; Department of Health Policy and Management, Johns Hopkins University Bloomberg School of Public Health, Baltimore, Maryland; Arnold School of Public Health, Health Services Policy and Management Department, University of South Carolina, Columbia; Crown Family School of Social Work, Policy, and Practice, The University of Chicago, Illinois

## Abstract

**IMPORTANCE:**

Medicaid is a key policy lever to improve opioid use disorder treatment, covering approximately 40% of Americans with opioid use disorder. Although approximately 70% of Medicaid beneficiaries are enrolled in comprehensive managed care organization (MCO) plans, little is known about coverage and prior authorization (PA) policies for medications for opioid use disorder (MOUD) in these plans.

**OBJECTIVE:**

To compare coverage and PA policies for buprenorphine, methadone, and injectable naltrexone across Medicaid MCO plans and fee-for-service (FFS) programs and across states.

**DESIGN, SETTING, AND PARTICIPANTS:**

This cross-sectional study analyzed MOUD data from 266 Medicaid MCO plans and FFS programs in 38 states and the District of Columbia in 2018.

**MAIN OUTCOMES AND MEASURES:**

For each medication, the percentages of MCO plans and FFS programs that covered the medication without PA, covered the medication with PA, and did not cover the medication were calculated, as were the percentages of MCO, FFS, and all (MCO and FFS) beneficiaries who were covered with no PA, covered with PA, and not covered. In addition, MCO plan coverage and PA policies were mapped by state. Analyses were conducted from January 1 through May 31, 2022.

**RESULTS:**

Coverage and PA policies were compared for MOUD in 266 MCO plans and 39 FFS programs, representing approximately 70 million Medicaid beneficiaries. Overall, FFS programs had more generous MOUD coverage than MCO plans. However, a higher percentage of FFS programs imposed PA for the 3 medications (47.0%) than did MCOs (35.9%). Furthermore, although most Medicaid beneficiaries were enrolled in a plan that covered MOUD, 53.2% of all MCO- and FFS-enrolled beneficiaries were subject to PA. Results also showed wide state variation in MCO plan coverage and PA policies for MOUD and the percentage of Medicaid beneficiaries subject to PA.

**CONCLUSIONS AND RELEVANCE:**

This cross-sectional study found variation in MOUD coverage and PA policies across Medicaid MCO plans and FFS programs and across states. Thus, Medicaid beneficiaries’ access to MOUD may be heavily influenced by their state of residency and the Medicaid plan in which they are enrolled. Left unaddressed, PA policies are likely to remain a barrier to MOUD access in the nation’s Medicaid programs.

## Introduction

Expanding access to treatment for opioid use disorder (OUD) remains a top public health priority. Opioid-related mortality reached an all-time high in 2021, exceeding 80 000 deaths.^1^ In 2020, approximately 2.5 million people met diagnostic criteria for OUD, but only 11.2% received any Food and Drug Administration (FDA)-approved medications for OUD (MOUD), including buprenorphine, methadone, and injectable naltrexone.^2^ Medications for OUD are associated with significant reductions in opioid use and fatal overdose, reductions in health care utilization related to opioid use, and increases in treatment retention rates.^3-7^

Medicaid is a key policy lever to improve OUD treatment because it covers approximately 40% of Americans with OUD. However, previous research has revealed wide state variation in benefits for OUD treatment across Medicaid fee-for-service (FFS) programs.^8,9^ Such variation in OUD treatment benefit design, including MOUD, may lead to differences in patient access to treatment and health outcomes for Medicaid enrollees with OUD.

Although the Substance Use-Disorder Prevention that Promotes Opioid Recovery and Treatment for Patients and Communities Act (SUPPORT Act) of 2018 requires that all Medicaid plans provide coverage for MOUD as of January 2020, it does not prohibit utilization management policies, such as prior authorization (PA).^10,11^ Prior authorization is primarily used by insurers as a cost control mechanism. This tool may be particularly important for administration of state Medicaid programs because Medicaid is consistently the most expensive item in state budgets. However, payers have also relied on PA to ensure appropriate use of care. For example, a recent study of 2 state Medicaid programs that completely removed PA on buprenorphine for OUD found an increase in buprenorphine prescribing in Illinois but no difference in prescribing after PA removal in California.^12^

This mixed result is important because although use of PA to control costs and limit unnecessary care can be effective, it can also unnecessarily restrict access to MOUD if its use denies necessary care or if PA decisions take too long and the opportunity for care engagement is lost. Many other studies identify the use of PA as a barrier to timely receipt of MOUD and continuity of care.^12-21^ Removal of PA in Medicare Part D plans, following an FDA labeling change for buprenorphine products and related Centers for Medicare & Medicaid Services (CMS) guidance,^22^ was associated with an increase in prescribing of buprenorphine and naloxone and a decrease in substance use disorder–related inpatient admissions and emergency department visits.^16^ Relatedly, removal of PA for MOUD was associated with a decrease in the likelihood of relapse among patients with OUD enrolled in a Medicare Advantage plan.^20^ Research also shows that specialty substance use disorder treatment programs are less likely to offer buprenorphine in states where Medicaid FFS programs impose PA on the medication.^13^ Prior authorization is also identified as a major barrier by buprenorphine prescribers serving Medicaid enrollees.^15,17^

Although there is documentation of widespread use of PA for MOUD in Medicaid FFS programs in 2017,^9^ little is known about MOUD coverage and PA policies in comprehensive Medicaid managed care organization (MCO) plans, which cover approximately 70% of all Medicaid beneficiaries.^23^ A comparison of PA policies in Medicaid FFS programs and MCO plans in 3 states found variation in the use of PA for MOUD,^24^ suggesting that additional research in this area is warranted. To address this gap in the literature, this study uses publicly available documentation from all Medicaid comprehensive MCOs to compare coverage and PA policies for buprenorphine, methadone, and injectable naltrexone across Medicaid MCO plans and FFS programs as well as across states.

## Methods

### Study Design and Data

In this cross-sectional study, we conducted a content analysis of all 266 Medicaid MCO plans that had active contracts in 38 states and the District of Columbia in 2018. We reviewed publicly available documentation on benefits and PA policies for buprenorphine, methadone, and injectable naltrexone. Documents reviewed were member handbooks, provider manuals, and prescription drug formularies. When multiple plans were available for different enrollee groups within a single MCO, we selected the largest comprehensive plan that served adult, nonelderly (aged 18-64 years) enrollees. The study was limited to comprehensive MCO plans and did not include primary care case management plans.

A search protocol (detailed in the Measures section) was used to collect MCO plan documents, which were reviewed and coded by trained research assistants. The research assistants met weekly with the research team to discuss coding decisions. All coding discrepancies were resolved through consultation with the research team. A doctorate-level research assistant was responsible for cleaning the data for analyses (M.M.W.). The University of Chicago’s institutional review board approved this study and waived the requirement for informed consent because non-human participant data were used. The study followed the Strengthening the Reporting of Observational Studies in Epidemiology (STROBE) reporting guideline.

### Measures

Buprenorphine, methadone, and injectable naltrexone were coded as covered if an MCO plan reported covering the medication in either its enrollee or provider handbook or if the medication was listed in its formulary. For each medication, plans were coded as imposing PA if the plan mentioned the need for PA in the member handbook, as a requirement for medication reimbursement in the provider handbook, or as a requirement in its formulary.

Coverage data for buprenorphine and injectable naltrexone were not specified for 1 MCO plan; there were no missing coverage data for methadone. Information on PA was not specified in 30 MCO plans for methadone, 5 plans for buprenorphine, and 1 plan for injectable naltrexone. We excluded from analyses all MCO plans for which coverage information was missing (1 plan for buprenorphine and injectable naltrexone) and/or PA information was not specified (30 plans for methadone, 5 plans for buprenorphine, and 1 plan for injectable naltrexone). Twelve states (11 for methadone and 1 for buprenorphine) with more than 50% of information not specified for PA were categorized as not specified. Of note, although we were unable to categorize these MCO plans’ PA policies, “not specified” is important because an individual with OUD who wanted to understand the details of a plan’s MOUD benefits would be unable to locate that information in the enrollee or provider handbook or formulary.

### Statistical Analysis

We compared MCO medication benefits with those specified by Medicaid FFS programs using FFS data from a survey of state Medicaid agencies conducted from May to December 2017.^9^ The current analysis was performed from January 1 through May 31, 2022.

Several measures of MOUD coverage and PA policies were constructed. First, for each medication (buprenorphine, methadone, and injectable naltrexone), we calculated the percentage of Medicaid MCO plans and FFS programs that (1) covered the medication without PA, (2) covered the medication with PA, and (3) did not cover the medication. Second, we used enrollment data from CMS^23^ to calculate the percentage of MCO, FFS, and all (MCO and FFS) beneficiaries who were (1) covered with no PA, (2) covered with PA, and (3) not covered. Third, to capture state variation in MOUD coverage and PA policies, we mapped the percentage of MCO plans covering each medication and the percentage of MCO plans requiring PA by state. Fourth, we mapped the percentage of all Medicaid beneficiaries (MCO and FFS) enrolled in a plan covering each medication and the percentage of all beneficiaries subject to PA for each medication by state. Analyses were performed using Stata, version 17.0 (StataCorp LLC).

## Results

### Medicaid MCO Plan vs FFS Program Coverage and PA Policies for MOUD

**Figure 1.**
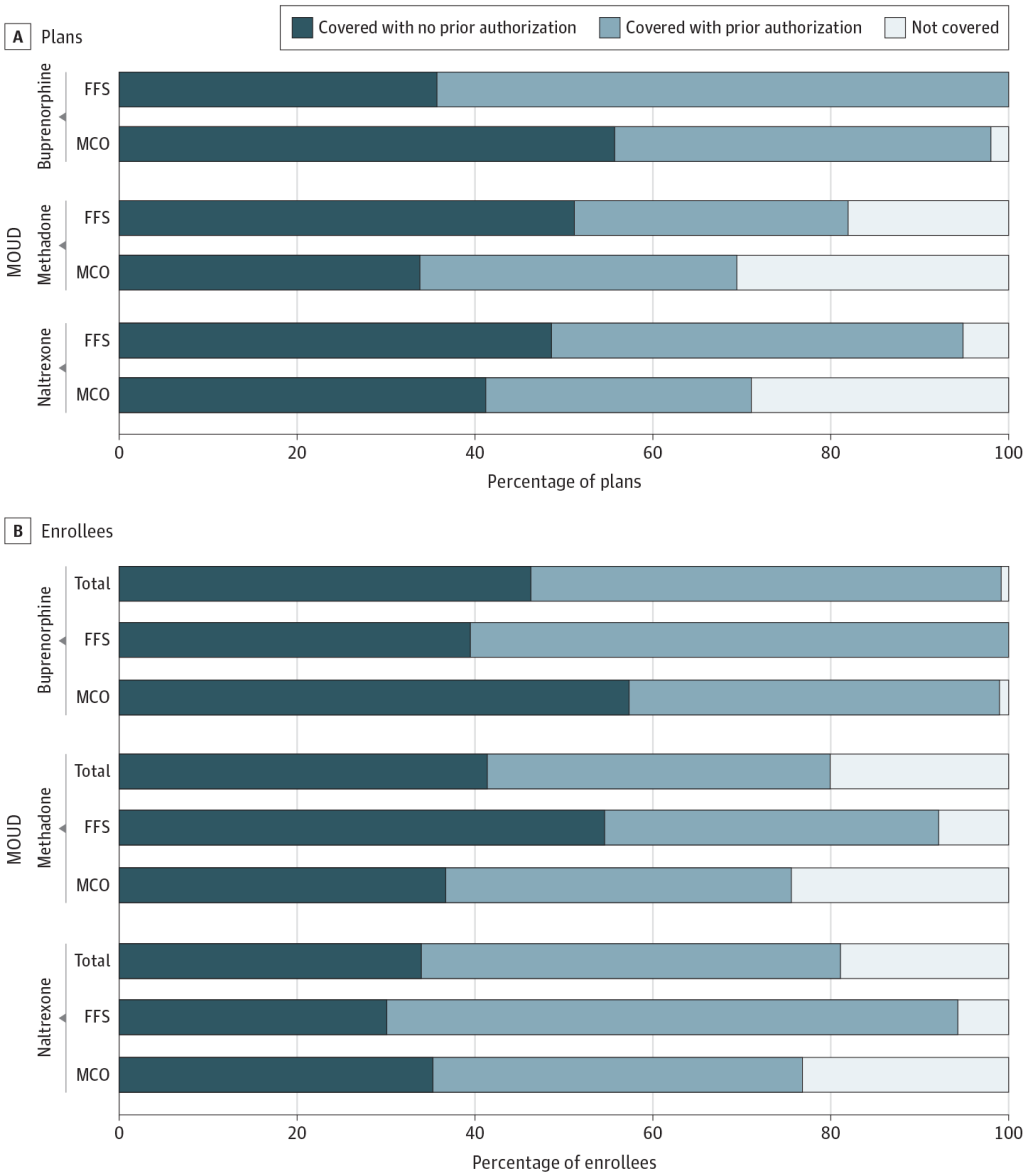
Percentage of Medicaid Managed Care Organization (MCO) Plans vs Fee-for-Service (FFS) Programs and Percentage of MCO vs FFS Enrollees With Coverage for Medications for Opioid Use Disorder (MOUD) With and Without Prior Authorization, 2018

This study examined coverage and PA policies in 266 MCO plans and 39 Medicaid FFS programs, representing approximately 70 million Medicaid beneficiaries in 2018. Overall, a lower percentage of MCO plans vs FFS programs covered MOUD in 2018 (Figure 1A). We found that all 39 Medicaid FFS programs (100%) covered buprenorphine, 32 (82.1%) covered methadone, and 37 (94.9%) covered injectable naltrexone. A lower percentage of MCO plans covered buprenorphine (255 [98.1%]), methadone (164 [69.5%]), and injectable naltrexone (188 [71.2%]).

Similarly, we found stark differences in the percentage of Medicaid beneficiaries enrolled in Medicaid FFS programs and MCO plans covering each medication (Figure 1B). Although almost all Medicaid beneficiaries, whether in an FFS program or MCO plan, had buprenorphine coverage (FFS, 17 061 820 [100%]; MCO, 51 526 807 [99.0%]), fewer MCO beneficiaries had coverage for methadone (FFS, 15 713 936 [92.1%]; MCO, 39 708 732 [75.6%]) and injectable naltrexone (FFS, 16 089 296 [94.3%]; MCO, 40 399 030 [76.8%]).

Turning to PA policies (Figure 1A), we found that a higher percentage of Medicaid FFS programs than MCO plans covered buprenorphine (25 [64.1%] vs 110 [42.3%]) and injectable naltrexone (18 [46.2%] and 79 [29.9%]) with a PA requirement. However, a slightly higher percentage of MCO plans than FFS programs covered methadone with a PA requirement (84 [35.6%] vs 12 [30.8%]). Overall, for the 3 medications, 47.0% of FFS programs imposed PA compared with 35.9% of MCO plans.

A higher percentage of FFS-enrolled beneficiaries than MCO-enrolled beneficiaries faced PA requirements for buprenorphine (FFS, 10 322 401 [60.5%]; MCO, 21 902 832 [41.7%]) and injectable naltrexone (FFS, 10 953 688 [64.2%]; MCO, 21 797 783 [41.5%]) (Figure 1B). A similar proportion of FFS- and MCO-enrolled beneficiaries had methadone coverage subject to PA (6 398 183 [37.5%] and 20 222 040 [38.5%], respectively).

Finally, of the total percentage of all Medicaid beneficiaries (MCO and FFS) facing PA requirements (Figure 1B), approximately 50% had buprenorphine coverage (36 811 310 [52.9%]) or injectable naltrexone coverage (32 775 288 [47.1%]) that required PA. In addition, almost 40% of all Medicaid beneficiaries had methadone coverage that required PA (27 069 187 [38.5%]). Overall, among beneficiaries with MOUD coverage, 53.2% were subject to PA.

### State Variation in Percentage of MCO Plans Covering MOUD

**Figure 2.**
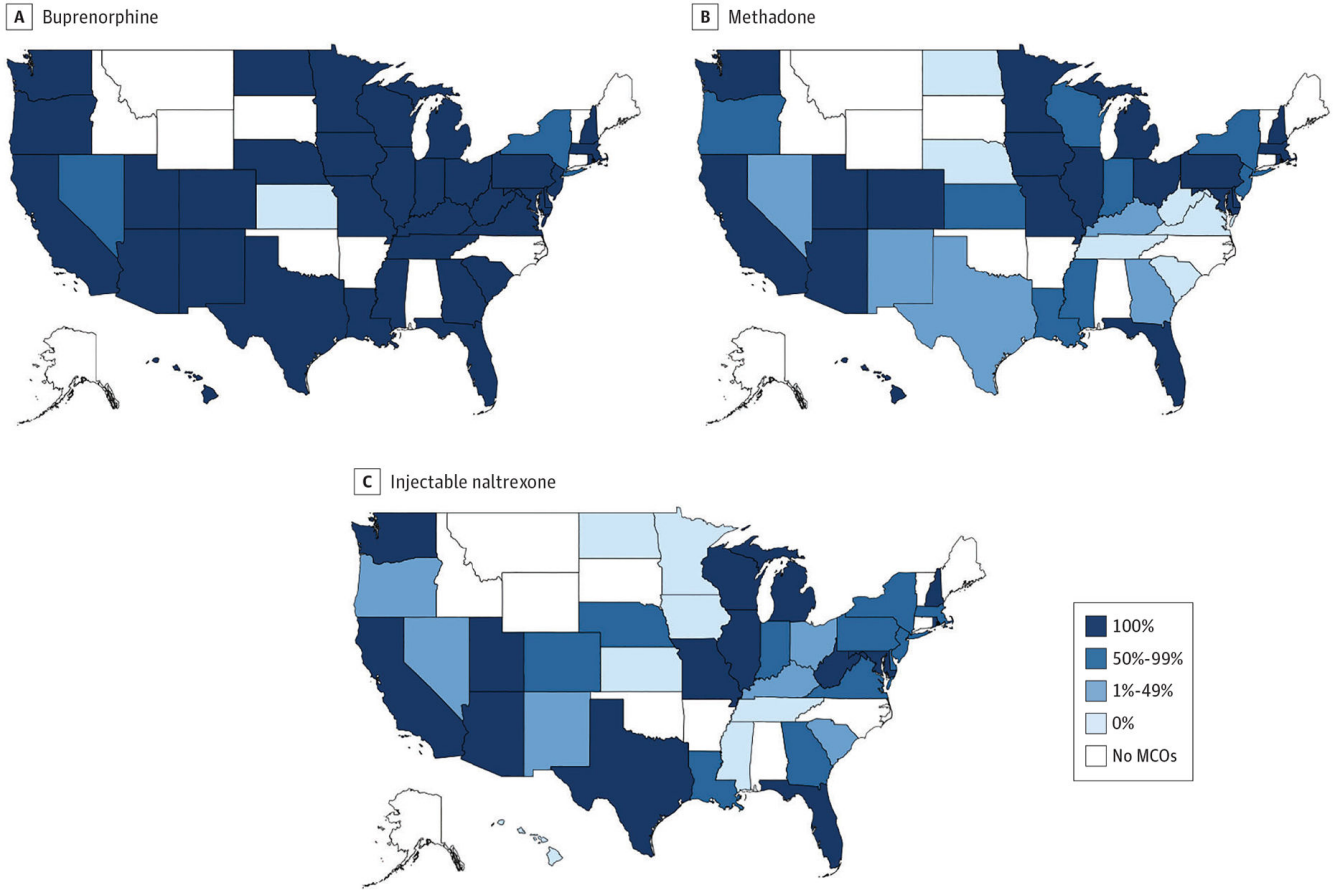
Percentage of Medicaid Managed Care Organization (MCO) Plans Covering Medications for Opioid Use Disorder by State, 2018

In 36 states (92.3%), all MCO plans covered buprenorphine, whereas no MCO plan covered buprenorphine in 1 state (2.6%) (Figure 2A). In contrast, in 19 states (48.7%), all MCO plans covered methadone, and in 6 states (15.4%), no MCO plans covered the medication (Figure 2B). Similarly, in 14 states (35.9%), all MCO plans covered injectable naltrexone, and in 7 states (18.0%), no plans covered the medication (Figure 2C).

### State Variation in Percentage of All Medicaid Beneficiaries Enrolled in Plans Covering MOUD

**Figure 3.**
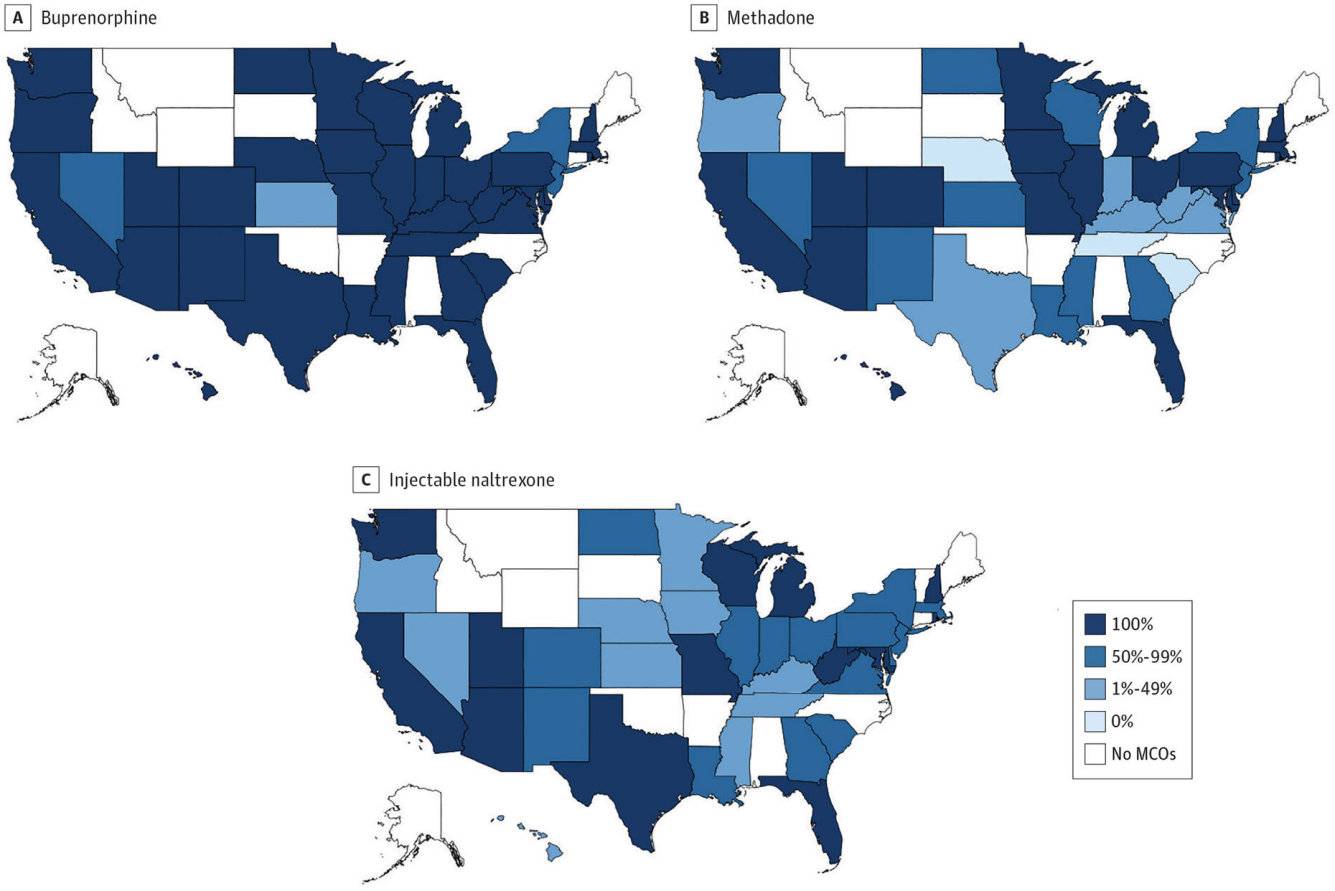
Percentage of All Medicaid Beneficiaries Enrolled in Fee-for-Service Programs and Managed Care Organization (MCO) Plans Covering Medications for Opioid Use Disorder by State, 2018

For Medicaid beneficiaries (MCO and FFS) enrolled in plans that covered MOUD, coverage was less generous for methadone and injectable naltrexone than buprenorphine (Figure 3). In 10 states (25.6%), less than 50% of beneficiaries were enrolled in plans covering methadone or injectable naltrexone, whereas there was only 1 state (2.6%) with less than 50% of beneficiaries enrolled in a plan covering buprenorphine.

### State Variation in Percentage of MCO Plans Requiring PA for MOUD

**Figure 4.**
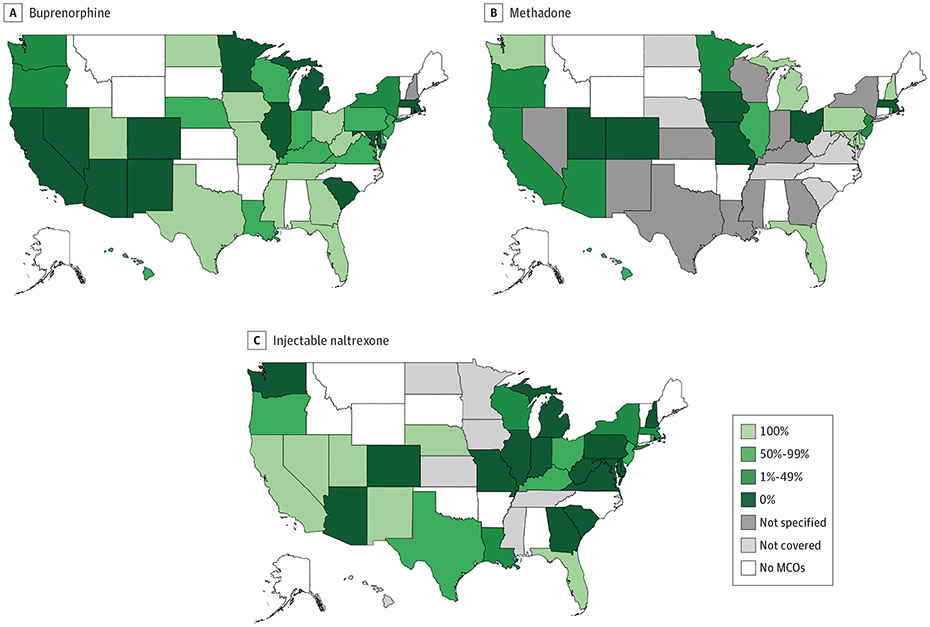
Percentage of Medicaid Managed Care Organization (MCO) Plans Requiring Prior Authorization by State, 2018

Prior authorization policies for buprenorphine were not specified in 1 state (Figure 4A). Among the remaining 37 states and the District of Columbia with PA information, all required PA for buprenorphine in 11 states (29.0%), whereas none required PA for the medication in 12 states (31.6%).

Prior authorization policies were not specified for methadone in 11 states (Figure 4B). Among the 22 states with MCOs that covered methadone and provided PA data, all MCO plans required PA for methadone in 7 states (31.8%), and none required PA for methadone in 6 states (27.3%). Among 32 states with MCO plans that covered injectable naltrexone (Figure 4C), all required PA in 6 states (18.8%), and none required PA in 15 states (46.9%).

### State Variation in Percentage of All Medicaid Beneficiaries Subject to PA for MOUD

**Figure 5.**
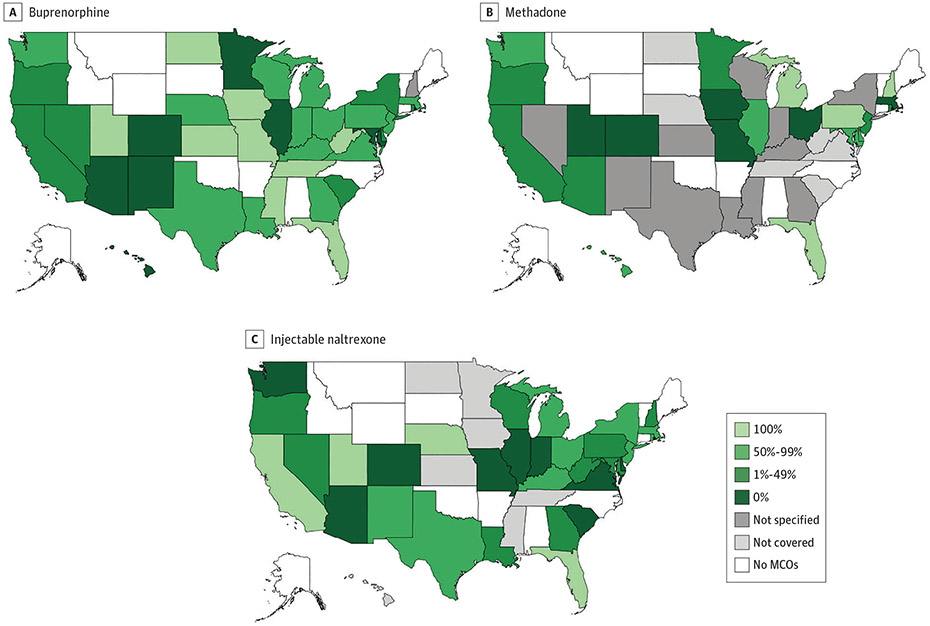
Percentage of All Medicaid Beneficiaries Enrolled in Fee-for-Service Programs and Managed Care Organization (MCO) Plans Subject to Prior Authorization by State, 2018

In 9 states (23.7%), all Medicaid (MCO and FFS) enrollees had buprenorphine coverage subject to PA (Figure 5A) vs 4 states (18.2%) for methadone (Figure 5B) and 4 states (12.5%) for injectable naltrexone (Figure 5C). No enrollees had buprenorphine coverage that required PA in 7 states (18.4%) (Figure 5A). In contrast, no enrollees had methadone coverage subject to PA in 6 states (27.3%) (Figure 5B), and none had injectable naltrexone coverage subject to PA in 9 states (28.1%) (Figure 5C).

## Discussion

The findings from this cross-sectional study fill an important gap in the literature on MOUD benefit designs in state Medicaid programs. To our knowledge, this study is the first on a national level to investigate MOUD coverage and PA policies for all 3 FDA-approved MOUDs in Medicaid MCO plans and FFS programs. The findings reveal key differences in both MOUD coverage and PA policies between MCO plans and FFS programs. Importantly, we also found that approximately one-half of all Medicaid beneficiaries (MCO and FFS) had MOUD coverage that required PA.

Overall, Medicaid FFS programs have more generous MOUD coverage; however, a majority of beneficiaries (approximately 70%) are enrolled in Medicaid MCO plans.^23^ In addition, although a higher percentage of state Medicaid FFS programs than MCO plans impose PA on MOUD, this affects a small proportion of all Medicaid enrollees (ranging from 12.1% of enrollees for methadone to 21.3% for injectable naltrexone) because the total number of beneficiaries enrolled in FFS programs is relatively small compared with enrollment in Medicaid MCO plans.^12^

The SUPPORT Act is expected to address inequities in coverage of MOUD across Medicaid MCO plans and FFS programs by requiring all plans to cover buprenorphine, injectable naltrexone, and methadone. However, the legislation does not address the use of PA and other utilization management strategies. Thus, it is possible that state Medicaid FFS programs and MCO plans could increase their use of such policies to reduce the costs associated with newly covering these medications. To date, compliance with the SUPPORT Act and whether all Medicaid plans now cover buprenorphine, methadone, and injectable naltrexone have not been studied. Importantly, the current study provides a baseline (before the SUPPORT Act) measure of MOUD coverage in both Medicaid FFS programs and MCO plans.

Since 2018, the District of Columbia and 8 states in our study have passed legislation that places some limits on the use of PA in their state Medicaid FFS programs for at least some MOUDs.^12,18^ Of these states, only 3 have completely removed PA policies for MOUD, and only 1 state law explicitly restricts the use of PA in Medicaid MCO plans (Colorado). However, in 2018, no comprehensive MCO plans in Colorado required PA. These differences in state approaches highlight variations in PA policies more generally. Prior authorization policies can be used for certain groups of enrollees, for certain dosages, or at different points along the treatment continuum.^21^ For example, some policies require PA only at the time of the initial prescription, whereas others also require PA at subsequent dosing periods. Such variations in PA policy could have important differential effects on the OUD cascade of care and related health outcomes.^25^ For example, policies that require PA at the time of the initial prescription may delay time to treatment initiation, whereas policies requiring PA beyond the initial dose may disrupt treatment, thereby possibly affecting retention in care and ultimately remission and recovery. Additional research is needed to document with greater specificity these variations among PA policies and then to determine whether variations in PA policies are associated with the OUD cascade of care.^21^

### Limitations

This study has several limitations. First, we compared FFS program survey data collected in the latter half of 2017 with MCO plan data for calendar year 2018. It is possible that some state Medicaid FFS programs may have altered their medication benefits during 2018. Second, PA information was not specified in 30 MCO plans for methadone and 5 for buprenorphine; thus, we were not able to include 11 states in our analyses of PA policies for methadone and 1 state for buprenorphine. However, because these data are not publicly available, this limitation also highlights the need for MCOs to disclose their PA policies. Third, our data predate important policy changes under the SUPPORT Act (which requires coverage of all FDA-approved MOUD by state Medicaid programs) and the enactment of some state laws restricting the use of PA for MOUD in Medicaid MCO plans. However, this study provides a baseline to assess the association of these policies with changes in coverage and PA policies in Medicaid FFS programs and MCO plans.

Fourth, we were unable to identify variation in PA policies beyond a yes/no classification. As described earlier, studies that investigated state legislation designed to place limits on PA in Medicaid found variation in these policies, particularly with regard to whether states have completely removed PA or partially removed PA for MOUD.^12,18^ This level of detail was not collected in the Medicaid FFS survey data used in this study and was not available in the majority of MCO plan documents we reviewed. Further, we did not examine why variation in financing policies exists across states and in FFS programs vs MCO plans. Differences in coverage and PA policies may be attributed to a range of factors, including differences associated with patient selection into plans, regulations governing FFS programs vs MCO plans, state political environments, and other state MOUD policies.

## Conclusions

Overall, the findings from this cross-sectional study suggest that Medicaid beneficiaries’ access to MOUD may be heavily influenced by their state of residence and the particular Medicaid plan in which they are enrolled. Thus, state Medicaid agencies should consider reviewing their contractual agreements with MCO plans to ensure appropriate coverage and use of PA that is consistent with best existing clinical guidance. In addition, CMS should consider issuing guidance to support the removal of PA for MOUD in all Medicaid plans (MCO and FFS) as it has done for the Medicare program. Finally, more states should consider passing legislation that limits the use of PA for MOUD in their Medicaid programs. Left unaddressed, PA policies are likely to remain a critical barrier to MOUD access in the nation’s Medicaid programs.

## Key Points

### Question

How do coverage and prior authorization (PA) policies for medications for opioid use disorder (MOUD) vary across Medicaid fee-for-service (FFS) programs and managed care organization (MCO) plans and across states?

### Findings

In this cross-sectional study of 266 Medicaid MCO plans and 39 FFS programs, FFS programs offered more generous MOUD coverage, but a higher percentage of FFS programs imposed PA than MCO plans. Although most Medicaid MCO and FFS beneficiaries were enrolled in a plan that covered MOUD, approximately 50% were subject to PA.

### Meaning

Despite concerns that PA policies are a barrier to MOUD access, these policies remain widespread in the nation’s Medicaid programs.
