# Error in Figure Titles

**DOI:** 10.1001/jamahealthforum.2022.4653

**Published:** 2022-11-18

In the Original Investigation titled “Coverage and Prior Authorization Policies for Medications for Opioid Use Disorder in Medicaid Managed Care,”^1^ published November 4, 2022, the titles for Figures 3 and 5 have been corrected to include mention of fee-for-service programs; the titles had originally only mentioned managed care organization plans, when these figures in fact report on both. This article has been corrected.^1^
